# Trends in self-rated health and association with socioeconomic position in Estonia: data from cross-sectional studies in 1996–2014

**DOI:** 10.1186/s12939-016-0491-9

**Published:** 2016-12-08

**Authors:** Mariliis Põld, Kersti Pärna, Inge Ringmets

**Affiliations:** 1 Estonian Health Insurance Fund, Lastekodu 48, 10144 Tallinn, Estonia; 2 Institute of Family Medicine and Public Health, University of Tartu, Ravila 19, 50411 Tartu, Estonia

**Keywords:** Self-rated health, Trends, Adults, Socioeconomic position, Estonia

## Abstract

**Background:**

Self-rated health (SRH) and socioeconomic position (SEP) as important determinants of health differences are associated with health and economic changes in society.

The objectives of this paper were (1) to describe trends in SRH and (2) to analyze associations between SRH and SEP among adults in Estonia in 1996–2014.

**Methods:**

The study was based on a 25–64-year-old subsample (*n* = 18757) of postal cross-sectional surveys conducted every second year in Estonia during 1990–2014. SRH was measured using five-point scale and was dichotomized to good and less-than-good. Standardized prevalence of SRH was calculated for each study year. Poisson regression with likelihood ratio test was performed for testing trends of SRH over study years. Age, nationality, marital status, education, work status and income were used to determine SEP. Logistic regression analysis was used to assess association between SRH and SEP.

**Results:**

The prevalence of dichotomized good self-rated health increased significantly over the whole study period with slight decrease in 2008–2010. Until 2002, good SRH was slightly more prevalent among men, but after that, among women. Good SRH was significantly associated with younger age, higher education and income and also with employment status among both, men and women. Good SRH was more prevalent among Estonian women and less prevalent among single men.

**Conclusions:**

There was a definite increase of good SRH over two decades in Estonia following economic downturn between 2008 and 2010. Good SRH was associated with higher SEP over the study period. Further research is required to study the possible reasons behind increase of good SRH, and it’s association with SEP among adults in Estonia.

## Background

**Fig. 1:**
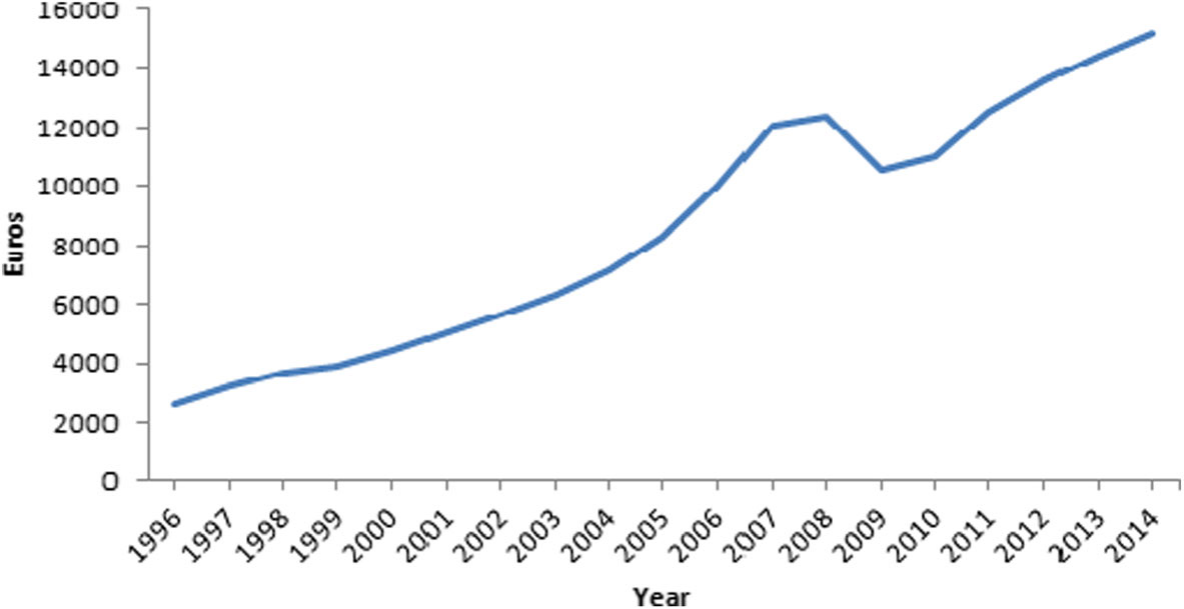
Gross domestic product (GDP) per capita 1996–2014

Health as a basic human right [1] is associated with personal and environmental factors. After the reindependence in 1991, societal and economic reforms brought major changes to the residents of Estonia. The year 1996 represented the time of overwhelming transition followed by economic stabilization and fast growth (1996–2004), joining both EU and NATO in 2004, continuing economic growth and slowdown (2005–2008), economic downturn in 2008–2010 and stabilization since 2010 [2]. Since 2006, Estonia is considered as a high income country, according to World Bank [3]. In terms of economic development, gross domestic product (GDP) increased from 2578 Euros per person in 1996 to 12353 in 2008 (Fig. 1) [4]. In 2009, the GDP per capita was about 15% lower (10600) followed by new increase (15186 in 2014).

**Fig. 2:**
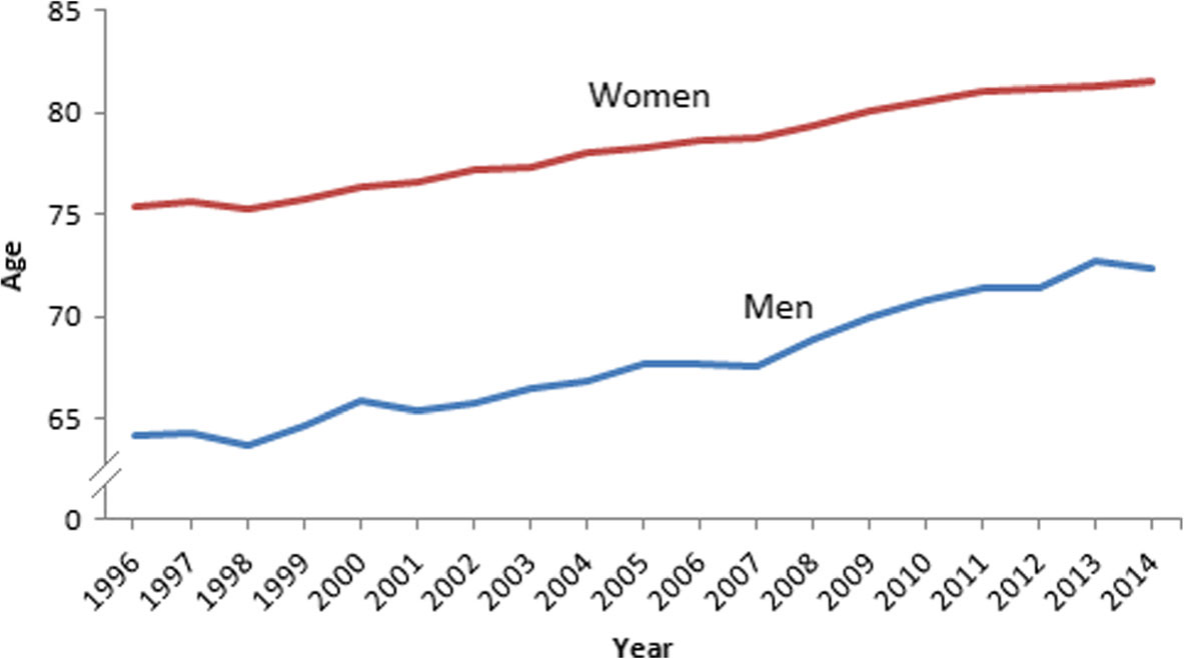
Life expectancy at birth, 1996–2014

At the same time life expectancy at birth increased steadily from 1996 (64.1 for men, 75.4 for women) to 2014 (72.3 for men, 81.5 for women) (Fig. 2) [5].

Among health measures besides of life expectancy, self rated health (SRH) can be used as an option which is recommended by WHO [6]. SRH is based on a single question asking people to rate their overall health status and has been shown to be reliable measure of individuals’ subjective health [6, 7]. According to previous survey results, the prevalence of good SRH varied greatly and was more prevalent among men in countries like Austria, France, Latvia, Norway, Poland, Russia, Armenia as well as in Estonia [8–13] but among women in Finland, Ireland and Great Britain [8]. Although most of the studies cited here were based on single-year data, there is less amount of studies concerning time trends in SRH. Some earlier studies have reported the worsening of SRH over recent decades, for example in USA [14, 15] and improvement of SRH in Finland, Lithuania and Russia [2, 16].

Sociodemographic and socioeconomic factors can predict how a person might rate their health [17, 18]. Factors like gender, age, nationality, marital status, place of residence, education, employment status, occupation and income are viewed as determinants of health differences and SEP [10, 11]. As shown in previous international studies, a higher SEP was related to better health [19, 20]. Adults with higher education [11, 21] and income [9, 10, 22] rated their health as good more often than people with lower education and income. Good SRH was more prevalent among employed adults when compared to respondents who were unemployed [2, 11]. Younger people rated their health as good more often than people in older age groups [9, 21, 23]. The prevalence of good SRH was higher among married or cohabited adults when compared to adults who were single, divorced or widowed [10, 23, 24]. When considering nationality, good SRH was more prevalent among main ethnicity in Estonia [10] and in Finland [2]. There is limited overview concerning trend analysis of SRH and association with SEP among adult population in Estonia over the last decades. In order to support priority-setting and evaluate the impact of health policies, evidence-based information is needed.

The objectives of this paper were (1) to describe trends in SRH and (2) to analyze associations between SRH and SEP among adults in Estonia in 1996–2014.

## Methods

### Data and sample

The study was based on data drawn from the cross-sectional postal survey of Health Behaviour among Estonian Adult Population conducted among 16–64-year-old adults in every second year. The survey started in Estonia in 1990 as part of the Finbalt Health Monitor project. The surveys were approved by the Tallinn Medical Research Ethics Committee. A random sample, stratified by age, gender and place of residence, of the Estonian population aged 16–64 was taken from the Population Registry. The methodology of survey of Health Behaviour among Estonian Adult Population is described in more detail elsewhere [25, 26].

In 1996–2002 the initial sample consisted of 2000 adults, in 2004–2014 the sample size was 5000 adults (Table 1). The crude response rate of the inital sample was the highest in 1996 (75.4%) and lowest in 2014 (51.5%). Crude and corrected response rates were calculated for the initial sample where data was available. The corrected response rate was calculated by exluding those persons from the sample who were ineligible (did not live at the address provided, no letter box available, not living in Estonia, had died). In 2004–2014, the crude and corrected response rates for this age group were similar to response rates of initial sample. This paper studied the population aged 25–64. The crude and corrected response rates were calculated (Table 1). The adults under 25-years-old were excluded because of their possibly uncompleted education.

**Table 1:** Sample size, crude and corrected response rates of initial sample (16–64-year-olds) and study sample (25–64-year-olds) by study year, 1996–2014

Year	Sample size	Initial survey sample		Study subsample	
		Response rate		Response rate	
		Crude	Corrected	Crude	Corrected
1996	2000	75.4	-	-	-
1998	2000	66.1	-	-	-
2000	2000	68.8	-	-	-
2002	2000	66.9	-	-	-
2004	5000	61.5	63.4	62.3	64.0
2006	5000	57.3	59.2	58.3	60.1
2008	5000	60.1	62.2	60.4	62.6
2010	5000	60.5	62.3	61.6	63.6
2012	5000	59.4	62.0	60.6	63.4
2014	5000	51.5	53.3	53.2	55.1

 The subsample consisted of 18757 adults including 7660 men (40.8%) and 11097 women (59.2%) in 1996–2014 (Table 2).

**Table 2:** Study sample^a^ of 25–64-year-old men and women by study year, 1996–2014

Year	Men		Women		Total
	N	%	N	%	N
1996	526	43.0	696	57.0	1222
1998	455	42.1	626	57.9	1081
2000	428	39.8	648	60.2	1076
2002	416	39.7	633	60.3	1049
2004	1023	41.0	1415	59.0	2438
2006	884	38.3	1423	61.7	2307
2008	1017	42.2	1393	57.8	2410
2010	999	40.4	1473	59.6	2472
2012	1017	41.1	1460	58.9	2477
2014	895	40.2	1330	59.8	2225
Total	7660	40.8	11097	59.2	18757

^a^Number of persons for whom the SRH question was completed in the questionnaire

### Variables

SRH was measured by a single question. Until 2002, the wording of the question was: ‘How would you currently assess your general state of health?’. From 2004, the question was ‘How would you assess your current state of health?’. There were five response categories: good, rather good, average, rather bad, bad. The responses were dichotomized to good (good/rather good) and less-than-good (average/rather bad/bad) SRH.

To describe SEP, the variables age, nationality, marital status, education, employment status and income were used and categorized as described below.

Age was measured in full years and analyzed in four age-groups: 25–34, 35–44, 45–54, 55–64. Nationality referred to self-determined national identity and data was categorized into two groups: Estonians and non-Estonians. Marital status was categorized into married/cohabiting; single, divorced/widowed. Education was based on the highest completed education levels and designated as follows: basic; secondary; higher education. Economic activity was measured using employment data and categorized to four groups: currently employed; unemployed; retired and not working; other (student, homemaker, conscripts). Income was determined by average monthly income per family member. Data was categorized into four groups based on quartiles that were calculated separately for each year and designated as follows: I (the lowest); II; III; IV (the highest).

### Statistical analysis

The data was analyzed separately for men and women. Age-standardized prevalences of SRH for each study year were calculated, using the European standard population [27]. Poisson regression with likelihood ratio test was performed for testing trends of SRH over study years. Logistic regression analysis was used to assess association between SRH and SEP. Firstly, logistic regression analysis was conducted separately for each study year to analyse socioeconomic differences by year (data not shown). Secondly, as the associations between SRH and SEP were similar throughout the study years with minor distinctions, logistic regression was used for the pooled data. In this model, SRH was used as a dependent variable and study year, age group, nationality, marital status, education, employment status and income as explanatory variables. Crude and adjusted odds ratios (OR) for good health and 95% confidence intervals (95% CI) were calculated.﻿ ORs were adjusted for all the variables.

Questionnaires which lacked information about SRH (*n* = 142) were excluded from the analysis. A total of 18757 questionnaires (7660 men and 11097 women) were used in the analysis. Questionnaires with missing information concerning SEP were excluded from the logistic regression analysis. A total of 7211 questionnaires for men and 10465 for women were used in the model.

Statistical package Stata 12 was used to analyze data.

## Results

### Trends in self-rated health in 1996–2014

**Fig. 3:**
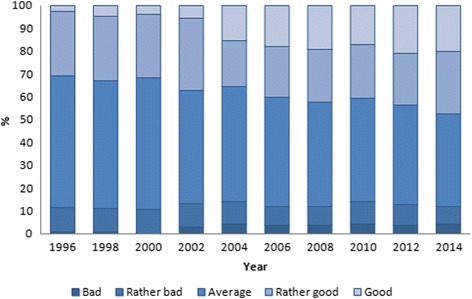
Age-standardized prevalence of SRH (*p* < 0.0001 for trend) among men in Estonia, 1996–2014

Based on five-point-scale the age-standardized prevalence of SRH changed significantly among men and women during the study period (*p* < 0.0001). Among men, good health increased from 2.7% in 1996 to 20.1% in 2014 (Fig. 3). The prevalence of average health ranged from 57.4 to 40.8%. Bad assessments were given by 0.7 to 4.1% of men during the whole study period.

**Fig. 4:**
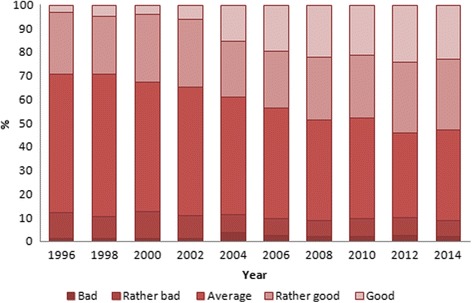
Age-standardized prevalence of SRH (*p* < 0.0001 for trend) among women in Estonia, 1996–2014

Among women, the prevalence of good health increased the most (ranging from 3.1 to 22.7%) in 1996–2014 (Fig. 4). Average SRH was reported by 58.9% of women in 1996 and 38.3% in 2014. Bad assessments were given by 1.1% in 1996, 3.8% in 2004 and 2.0% in 2014.

**Fig. 5:**
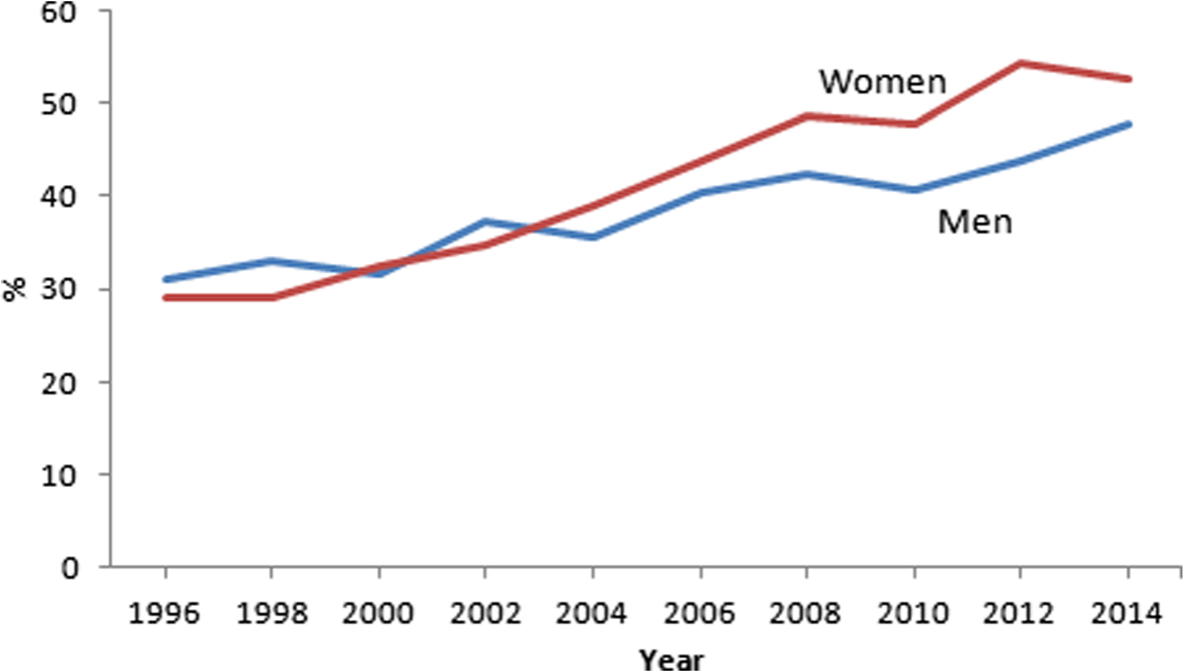
Age-standardized prevalence of good SRH (*p* < 0.0001 for trend) among men and women in Estonia, 1996–2014

Based on dichotomized scale of SRH, the age-standardized prevalence of good health increased significantly during the whole study period (*p* < 0.0001) (Fig. 5). In 1996, the age standardized prevalence was 29.1% among women and 31.1% among men, but in 2014, the prevalence was 52.7 and 47.7%, respectively. Until 2004, good SRH was more prevalent among men but since then women assessed their health as good more often. There was a slight decrease in prevalence of good SRH among men and women after the year 2008, followed by new increase since 2010.

### Associations between SRH and SEP

Compared to the first study year, good health was significantly higher in 2006–2014 among men and in 2004–2014 among women (Table 3). SRH was associated with almost all selected socioeconomic factors. Compared to the oldest age group of men and women, the odds of having good SRH was higher in all younger age groups being the highest in the youngest age group (25–34-years-old) (OR = 5.47, 95% CI 4.59–6.51 for men and OR = 4.99, 95% CI 4.32–5.78 for women). Odds to rate their health as good was 1.88 times higher among Estonian women than among non-Estonian women. After adjustment, nationality appeared not to be associated with SRH among men. Compared to married/cohabiting men, the odds of having good SRH was lower among single men (OR = 0.79, 95% CI 0.67–0.93). After adjustment, no significant association was found between SRH and marital status among women. Adults with secondary and higher education rated their health as good significantly more often than adults with basic education (for higher education OR = 3.51, 95% CI 2.89–4.28 among men and OR = 3.27, 95% CI 2.69–3.98 among women). Compared to the employed respondents, the odds of rating their health as good was significantly lower among unemployed and retired (for unemployed men OR = 0.70, 95% CI 0.57–0.87 and for women OR = 0.70, 95% CI 0.57–0.87). After adjustment, SRH appeared not to be associated with being a student, homemaker, recruited (subgroup ‘others’). A significant association was found between SRH and income. Compared to the lowest income group, the odds of rating their health good was about two times higher among adults in the highest income group (OR = 2.44, 95% CI 2.03–2.94 for men and OR = 2.21, 95% CI 1.91–2.57 for women).

**Table 3:** Odds ratios (OR) for good SRH in Estonia, 1996–2014^a^

Variable	Men			Women		
	OR (95% CI)					
	N	Crude	Adjusted^b^	N	Crude	Adjusted^b^
Year						
1996	526	1	1	696	1	1
1998	454	1.06 (0.81–1.40)	0.93 (0.69–1.27)	626	0.98 (0.77–1.24)	0.87 (0.66–1.14)
2000	428	0.97 (0.73–1.27)	0.97 (0.70–1.33)	648	1.09 (0.86–1.38)	1.06 (0.81–1.39)
2002	416	1.22 (0.93–1.60)	1.26 (0.93–1.72)	633	1.22 (0.97–1.54)	1.16 (0.90–1.51)
2004	1023	1.21 (0.97–1.52)	1.11 (0.86–1.43)	1415	**1.42 (1.17**–**1.73)**	**1.37 (1.10**–**1.71)**
2006	884	**1.37 (1.09**–**1.72)**	**1.40 (1.08**–**1.82)**	1423	**1.78 (1.47**–**2.17)**	**1.69 (1.35**–**2.10)**
2008	1017	**1.55 (1.24**–**1.94)**	**1.54 (1.20**–**1.99)**	1393	**2.21 (1.82**–**2.70)**	**2.15 (1.73**–**2.68)**
2010	999	**1.44 (1.15**–**1.80)**	**1.52 (1.18**–**1.96)**	1473	**2.06 (1.70**–**2.50)**	**2.07 (1.67**–**2.58)**
2012	1017	**1.57 (1.26**–**1.96)**	**1.66 (1.29**–**2.14)**	1460	**2.57 (2.12**–**3.12)**	**2.71 (2.17**–**3.37)**
2014	895	**1.86 (1.48**–**2.33)**	**1.86 (1.44**–**2.41)**	1330	**2.34 (1.92**–**2.90)**	**2.36 (1.89**–**2.95)**
Age group						
55–64	1829	1	1	2920	1	1
45–54	1943	**1.73 (1.49**–**2.01)**	**1.41 (1.19**–**1.67)**	2961	**1.92 (1.71**–**2.16)**	**1.60 (1.40**–**1.83)**
35–44	1958	**3.25 (2.81**–**3.76)**	**2.73 (2.31**–**3.23)**	2672	**3.64 (3.24**–**4.09)**	**3.12 (2.72**–**3.58)**
25–34	1930	**6.48 (5.59**–**7.51)**	**5.47 (4.59**–**6.51)**	2544	**6.45 (5.72**–**7.27)**	**4.99 (4.32**–**5.78)**
Nationality						
Non-Estonian	2232	1	1	3504	1	1
Estonian	5396	**1.26 (1.14**–**1.40)**	1.08 (0.96–1.22)	7560	**2.06 (1.89**–**2.24)**	**1.88 (1.70**–**2.07)**
Marital status						
Married, cohabited	5796	1	1	7444	1	1
Single	1069	1.06 (0.93–1.21)	**0.79 (0.67**–**0.93)**	1204	**1.19 (1.05**–**1.35)**	0.88 (0.77–1.02)
Divorced, widowed	752	**0.57 (0.48**–**0.67)**	0.89 (0.73–1.08)	2401	**0.57 (0.52**–**0.63)**	0.95 (0.85–1.06)
Education						
Basic	1300	1	1	1121	1	1
Secondary	4777	**2.38 (2.06**–**2.76)**	**1.82 (1.54**–**2.16)**	6766	**2.65 (2.26**–**3.11)**	**1.88 (1.57**–**2.26)**
Higher	1551	**5.38 (4.54**–**6.37)**	**3.51 (2.89**–**4.28)**	3154	**6.44 (5.45**–**7.60)**	**3.27 (2.69**–**3.98)**
Employment status						
Working	5838	1	1	7873	1	1
Unemployed	700	**0.42 (0.35**–**0.50)**	**0.70 (0.57**–**0.87)**	606	**0.44 (0.36**–**0.52)**	**0.70 (0.57**–**0.87)**
Retired	779	**0.12 (0.10**–**0.16)**	**0.37 (0.28**–**0.48)**	1224	**0.17 (0.14**–**0.20)**	**0.53 (0.43**–**0.65)**
Other	154	**0.56 (0.40**–**0.80)**	0.67 (0.44–1.01)	1131	**1.32 (1.17**–**1.50)**	1.13 (0.97–1.31)
Income (quartiles)						
I (the lowest)	1180	1	1	1811	1	1
II	1842	**1.35 (1.14**–**1.60)**	**1.37 (1.14**–**1.66)**	2985	1.02 (0.90–1.15)	1.09 (0.95–1.26)
III	2137	**2.11 (1.80**–**2.50)**	**1.71 (1.42**–**2.06)**	3201	**1.60 (1.42**–**1.81)**	**1.49 (1.29**–**1.72)**
IV (the highest)	2317	**4.15 (3.54**–**4.87)**	**2.44 (2.03**–**2.94)**	2827	**3.01 (2.66**–**3.41)**	**2.21 (1.91**–**2.57)**

^a^ Statistically significant associations (*p* < 0.05) are marked in bold

^b^ Adjusted for all other variables in the table

## Discussion

The study analyzed trends in SRH, and associations between SRH and SEP among 25–64-year-old adults in Estonia over the period of 1996–2014.

The main findings from the study were, first, that prevalence of good self-rated health increased over the whole study period with only a slight decrease between 2008 and 2010. Second, until 2002, good SRH was slightly more prevalent among men. After that, women rated their health as good more often. Third, SRH was associated with higher SEP from 1996 to 2014 throughout the whole study period.

### Strengths and limitations

The survey of Health Behaviour among Estonian Adult Population presents a great, and also the only opportunity to analyze self-rated health over more than two decades in Estonia. It is considered as a strength of the study, that the survey design and methodology have remained largely the same across the study period*.*

However, this study has several limitations that need to be considered. The survey was conducted as postal questionnaire and used self-reported data. Possible lower participation of adults with lower SRH or SEP has to be considered [28–30]. Another limitation could be related to the significantly smaller sample size until 2002. Although the response rate was acceptable for a population based study, power to detect significant differences could be affected by the small size of certain groups. The crude response rates ranged from 75.4 to 51.5% and declined across the survey years. Late response and item nonresponse in the Finbalt Health Monitor survey (including data from Estonia) has been analyzed earlier [29] and by assuming that nonrespondents were similar to late respondents, the authors concluded that the response bias could be minimal. Dichotomizing of SRH as good and less-than-good was done assuming that respondents who rate their health as average are feeling not healthy [31–33]. The categorization can affect the results as it is not definite to which group the ‘average’ is more similar to [32], however, a sensitivity analysis using different categorization for SRH (data not shown) demonstrated similar associations with SEP. Moreover, it has been reported that whether SRH was categorized to two groups or analyzed based on five point scale, the results were similar [2]. Despite these shortcomings, several inferences can be drawn.

### Trends in self rated health in Estonia

Based on five-point-scale, the most prevalent response of SRH was ‘average’ in every study year. Similar findings have been reported based on previous studies in Estonia [10, 11]. At the same time the prevalence of average SRH decreased one and half times over the study period being 40.8% for men and 38.3% for women in 2014. According to the earlier studies Eastern Europeans tend to choose middle categories more often when assessing their own health [11, 34]. In the current study, the prevalence of highest and lowest health ratings increased in 1996–2014, showing a remarkable rise from 2004. It has been reported that in recent years people are better at assessing their health [35].

The age standardized prevalence of dichotomized good SRH increased significantly over the study period. In 2014, the age-standardized prevalence of good SRH was 47.7% among men and 52.7% among women. Until 2002 the prevalence of good SRH was higher among men, after that, women rated their health as good more often. Earlier international studies have shown good SRH to be more prevalent among men in countries like Greece, Italy, Latvia, Armenia, Russia and elsewhere [8, 12, 13, 21]. However, there were a few countries, for example Finland and Ireland, where prevalence of good SRH was higher among women [8]. Possible reasons behind the exchange of positions in good SRH could be related to sharper increase of prevalence of women with higher education compared to men in Estonia. In the current study sample, the prevalence of higher education among women was 17.4% in 1996 but 35.6% in 2014 showing a steady rise over the years. For men, the prevalence of higher education was 16.7% in 1996 and 27.2% in 2014. The increase of prevalence of higher education among 25–64-year-old general population was similar according to Estonian census data from 2000 to 2011 [36, 37].

The results of the present study showed that compared to 2008 the prevalence of good SRH was lower in 2010. This finding is similar to results published earlier [2]. The period of 2008–2010 has been described as an economic downturn in Estonia and elsewhere [38–40]. When comparing GDP data before and after the recession, among Baltic countries the recovery from recession was overall similar in Estonia, Latvia and Lithuania [41]. The GDP in Estonia dropped notably after 2008 and regained pre-recession position only since 2012 [4]*.* Concerning economic recession, it has been suggested that for high-income countries it is unlikely that the recession will have any major overall negative health effects (e.g. on life expectancy or causes of death) [42]. In low-income countries the global economic recession could have negative effect related mainly to the lack of accumulated wealth and social protection support [42, 43]. There were no significant changes in life expectancy in Estonia when comparing the periods before 2008 to the economic dowturn and period after that as life expectancy increased among men and women during the whole study period [5]. Despite of this fact, the results from present study, however, indicated that economic decline in 2008–2010 might have had a negative effect on the SRH of adults in Estonia. When comparing to the neighbouring countries, a similar slight decline was reported in self-rated health in Lithuania in 2008–2010 [2].

### Association between SRH and SEP

Association between good SRH and SEP in this study were stable over the period of almost two decades in 1996–2014 being similar to the results published earlier [2, 10, 11, 23, 44]. Good SRH was significantly associated with younger age, higher education and income and also with employment status for both, men and women. Good health was more prevalent among Estonian women compared to non-Estonian women and lower among single men compared to married and cohabited men.

Younger age groups had higher odds to assess their health as good. These findings were in accordance with the results from previous studies [10, 11, 20, 28]. Among women, the odds of having good SRH were higher for Estonians when compared to non-Estonians. After adjustment, good SRH appeared not to be associated with nationality among men. Thus, ethnic differences in SRH presented a clear association only among women in this study. In earlier studies describing data from Estonia, controversial results were reported. Some studies showed that compared to non-Estonians, Estonians were more likely to rate their health as good [10, 23], but some studies found associations between SRH and nationality only among women [2]. In the present study sample, among non-Estonians, 26.3% of men and 22.9% of women rated their health as good (data not shown). It should be noted that transformation processes in Estonia has been particularly hard on non-Estonians [10, 11] and for example mortality rates are higher among this group [45].

Compared to the married or cohabited men, the odds of having good SRH were lower among single men. Previous studies have shown the opposite results – the odds of having average or lower SRH were lower among single men [23]. However, studies concerning risk behaviours like smoking and alcohol consumption reported that risk behaviours are more distributed among single men.

Compared to adults with basic and secondary education, the odds of having good SRH were more than three times higher among men and women with higher education. Higher education has been related to good SRH in studies in Estonia [2, 10, 11] and for example in Finland, Scotland, Belgium and the United States of America [20, 21]. Education is considered a key determinant of health as it underlies the possibilities of having better job and higher income [46, 47]. In terms of employment, the chances of having good SRH were significantly lower among the unemployed and retired respondents when compared to the employed adults in the current study. When interpreting the findings of this study, it has to be noted that the reasons for not working were not examined here. Compared to the adults in the lower income groups, the odds of having good SRH were higher among respondents in higher income groups. Income as a health determinant is related to availability of healthier choices. Employment and higher income have been related to better SRH in several previous studies in Estonia, Finland and worldwide [10, 11, 23, 48].

## Conclusions

This paper provided, on one hand, a new information concering a definite increase of good SRH over two decades following economic downturn in Estonia. Men assessed their health as good more often until 2004 but since then the prevalence of good SRH was higher among women. On the other hand, the association between good SRH and higher SEP was persistent over the whole study period. This study provides evidence-based information that could support identifying risk groups to decrease health inequalities in Estonia. A more in-depth analysis is required to determine the possible reasons behind increase of good self-rated health among adults in Estonia.

## Abbreviations

SEP: Socioeconomic position
SRH: Self-rated health
